# Calcium homeostasis behavior and cardiac function on left ventricular remodeling by pressure overload

**DOI:** 10.1590/1414-431X202010138

**Published:** 2021-02-12

**Authors:** I.F.S. Mazeto, K. Okoshi, C.F.S.M.P. Silveira, P.G. Sant'Ana, V.L. da Silva, G.A.F. Mota, S.L.B. de Souza, D.F. Vileigas, C.R. Padovani, A.C. Cicogna

**Affiliations:** 1 Departamento de Infectologia, Dermatologia, Diagnóstico por Imagem e Radioterapia, Faculdade de Medicina de Botucatu, Universidade Estadual Paulista, Botucatu, SP, Brasil; 2 Departamento de Clínica Médica, Faculdade de Medicina de Botucatu, Universidade Estadual Paulista, Botucatu, SP, Brasil; 3 Departamento de Bioestatística, Instituto de Biociências, Universidade Estadual Paulista, Botucatu, SP, Brasil

**Keywords:** Aortic stenosis, Diastolic dysfunction, Calcium-handling proteins, Rats, Pressure

## Abstract

Sarcoplasmic reticulum Ca^2+^-ATPase (SERCA2a) and sarcolemmal Na^+^/Ca^2+^ exchanger (NCX1) structures are involved in heart cell Ca^2+^ homeostasis. Previous studies have shown discrepancies in their function and expression in heart failure. The goal of this study was to evaluate heart function and hypertrophied muscle Ca^2+^-handling protein behavior under pressure overload. Twenty male Wistar rats were divided into two groups: Aortic stenosis (AoS), induced by a clip placed at the beginning of the aorta, and Control (Sham). After 18 weeks, heart function and structure were evaluated by echocardiogram. Myocardial function was analyzed by isolated papillary muscle (IPM) at basal condition and Ca^2+^ protein functions were evaluated after post-pause contraction and blockage with cyclopiazonic acid in IPM. Ca^2+^-handling protein expression was studied by western blot (WB). Echocardiogram showed that AoS caused concentric hypertrophy with enhanced ejection fraction and diastolic dysfunction inferred by dilated left atrium and increased relative wall thickness. IPM study showed developed tension was the same in both groups. AoS showed increased stiffness revealed by enhanced resting tension, and changes in Ca^2+^ homeostasis shown by calcium elevation and SERCA2a blockage maneuvers. WB revealed decreased NCX1, SERCA2a, and phosphorylated phospholambam (PLB) on serine-16 in AoS. AoS had left ventricular hypertrophy and diastolic dysfunction compared to Sham; this could be related to our findings regarding calcium homeostasis behavior: deficit in NCX1, SERCA2a, and phosphorylated PLB on serine-16.

## Introduction

Cardiac remodeling (CR) can be defined as changes in genetic, molecular, cellular, and interstitial expression, which can manifest as modifications in heart size, shape, and function as a response to specific aggressions, such as ischemia, inflammation, genetic mutations, and volume or pressure overload (1). CR is time-dependent and it can cause damage in the long term, representing an important risk factor for the development of ventricular dysfunction and heart failure (HF) (2,3).

There are several experimental models for studying CR, one being the pressure overload model (4–12). Pulmonary artery stenosis (7,8), abdominal aortic stenosis (9,10), and supravalvar aortic stenosis (AoS) (1,3,5,6) are examples of interventions used to study this condition. AoS has been used to promote the gradual development of left ventricular hypertrophy in young rats (1,3,5,6), with the effects appearing as weeks go by (3,5,11,12).

An important component of cardiac muscle contraction is Ca^2+^ homeostasis. In brief, this ion, which is present in the bloodstream through diet and bone balance, enters the cell through L-type channels and is stored in the sarcoplasmic reticulum until depolarization releases it to the muscle fibers through ryanodine receptors (RyR). Afterwards, with cell repolarization, the ion is recaptured through the sarcoplasmic reticulum calcium ATPase (SERCA2a) at the expense of ATP hydrolysis (13–15). Phospholambam (PLB) is a protein that regulates SERCA2a, increasing its function when it is phosphorylated (16). The concentration of Ca^2+^ in cytosol is closely controlled, and transporters such as Na^+^-Ca^2+^ exchanger (NCX1) are very important in maintaining cell gradient.

Regarding heart dysfunction, echocardiogram studies by our research group found that 18 weeks after AoS induction, rats develop left atrium (LA) remodeling, concentric left ventricular hypertrophy, diastolic dysfunction, and systolic function maintenance, improvement, or dysfunction (1,12).

Few studies have investigated Ca^2+^-transient proteins in experimental models of heart aggression with the above features, even though they should be considered part of the mechanisms involved in cardiac function loss. Some results in this area found that SERCA2a (9,10) is reduced in AoS (11,17–19). Also, a study using rats 6 weeks after AoS found that SERCA2a is related to functional changes (11). NCX1 was enhanced in hypertrophied hearts after infarction (20) but was at normal levels shortly after pressure overload (21). Røe et al. (22) found that rats presented enhanced SERCA and NCX1 activity after 6 weeks of aortic banding. They also concluded that LV complacency was altered regardless of changes in Ca^2+^ homeostasis. Rouhana et al. (23) showed that normal SERCA and NCX1 levels after 4 weeks increased phosphorylated PLB on threonine-17; Ca^2+^ was enhanced due to a lower extrusion through NCX1 and a larger leak from the sarcoplasmic reticulum through RyR. Hadipour-Lakmehsari et al. (24) reported that, after 8 weeks of transverse aortic constriction, RyR was reduced, SERCA enhanced, and PLB decreased after 2 weeks but was similar to Sham after 4 weeks.

Due to these divergent results on cardiac performance in left ventricular pressure overload remodeling and the function and expression of intracellular Ca^2+^-related proteins in non-transgenic animals, we decided to perform this study to evaluate cardiac function 18 weeks after AoS and to test whether functional variation of these proteins participates in hypertrophied muscle performance changes.

## Material and Methods

### Animal care

Twenty 30-day-old male Wistar rats weighting between 60 and 80 g were obtained from the Animal Center of Botucatu Medical School, São Paulo State University, UNESP (Brazil). All experiments and procedures were performed in accordance with the Guide for the Care and Use of Laboratory Animals published by the National Research Council (1996) and were approved by Botucatu Medical School Ethics Committee (protocol FMB-PE-30/2013). The rats were housed 4/cage in a controlled environment (12-h light/dark cycle starting at 6:00 am) and clean-air room temperature (23°C). They received food and water *ad libitum* and body weight (BW) was measured weekly.

### Experimental protocol

#### Groups

The two groups consisted of: AoS, 17 rats implanted with supravalvar bands, and Sham, 13 rats submitted to similar surgery but without band implant. During the study, 3 Sham and 5 AoS rats died. After applying the exclusion criteria below, each group contained 10 animals each.

#### Aortic stenosis surgery

Rats were anesthetized with intraperitoneal ketamine (50 mg/kg, *ip*) and xylazine hydrochloride (10 mg/kg, *ip*). A thoracotomy was performed and a silver band (0.6-0.7 mm internal diameter) was placed around the ascending aorta, as previously described (11,12,25–28). Exclusion criteria were evidence of heart failure.

#### In vivo functional and structural studies

All rats were submitted to echocardiographic evaluation 2-4 days before euthanasia using a Philips^®^ HDI 5000 apparatus (Philips Electronics North America Corporation, USA) to evaluate performance and the effects of surgery (29–35). Rats were anesthetized with a mixture of ketamine (50 mg/kg, *ip*) and xylazine hydrochloride (10 mg/kg, *ip*). Structural variables were measured: LV diastolic diameter (LVDD), LV diastolic posterior wall thickness (DPWT), relative wall thickness (RWT), left atrium diameter (LA), and aortic diameter (AO). LV function was assessed by heart rate (HR), ejection fraction (EF), midwall fractional shortening (FS), early and late diastolic mitral inflow velocities (E and A waves), E/A ratio, E wave deceleration time (EDT), and LV isovolumetric relaxation time (IVRT).

#### In vitro functional study: isolated papillary muscle

Rats were euthanized 18 weeks after surgery to harvest the heart, isolated papillary muscle (IPM), and the lungs. Animals were anesthetized with sodium pentobarbital (50 mg/kg, *ip*), euthanized, submitted to a thoracotomy, and the hearts were quickly removed and placed in oxygenated Krebs-Henseleit solution at 28°C. Papillary muscle was dissected carefully from the left ventricle, clipped at its edges, placed vertically in a chamber containing Krebs-Henseleit solution at 28°C, oxygenated with a mixture of 0.95 O_2_ and 0.5 CO_2_ (pH 7.38), and stimulated with two electrodes in the solution at a rate of 0.2 Hz. Papillary muscle performance was evaluated by a technique often used in our laboratory (11,36–38). The following basal parameters were measured from isometric contraction: peak developed tension (DT, g/mm^2^), resting tension (RT, g/mm^2^), time to peak tension (TPT, ms), maximum rate of tension development (+dT/dt, g/mm^2^ per s), and maximum rate of tension decline (−dT/dt, g/mm^2^ per s). The mechanical behavior of papillary muscle was evaluated at baseline condition with extracellular calcium (Ca^2+^) concentration of 2.5 mM. Then, the following inotropic maneuvers were performed: post-rest contraction, with Ca^2+^=0.5 mM; gradual Ca^2+^ elevation from 0.5 to 2.5 mM, to evaluate Ca^2+^ intake; and SERCA2a blockage by cyclopiazonic acid (CPA), as previously described (11,39).

The contractions were registered using a computer data system (AcqKnowledge^®^ MP100, Biopac Systems, Inc., USA).

Papillary muscle cross-sectional area (CSA, mm^2^) was calculated from muscle weight and length by assuming cylindrical uniformity and a specific gravity of 1.0. All force data were normalized for muscle CSA. Papillary muscles with CSA <0.5 and >1.5 mm^2^ were excluded from analysis.

#### Western blot study

Myocardial protein expression of SERCA2a, PLB, NCX1, RyR, and L-type Ca^2+^ channel in both groups were evaluated by western blot analysis according to the currently accepted procedure (38). Briefly, a total of 50 μg protein lysate was resolved by SDS-PAGE and transferred to a nitrocellulose membrane (Armsham Biosciences, USA) through transblot (semi-dry) to all proteins except L-type channel and ryanodine (wet transfer) with primary antibodies against SERCA2 ATPase (Affinity BioReagebts, USA), phospho-phospholambam (Affinity BioReagebts), phospho-phospholambam Ser16 and Thr17 (Badrilla, UK), exchanger Na^+^/Ca^2+^ (NCX1, Upstate, USA), calcium channel voltage gated alpha 1C (Chemicon International, USA), ryanodine receptor (Affinity BioReagents), and β-actin (Cell Signaling Technology #4967S, USA).

#### Post mortem morphological analysis

The presence of ventricular and atrial hypertrophy was determined by analyzing BW, tibia length (TB), and the ratio between left ventricle (LV), right ventricle (RV), atrium (AT), and total heart (TH) per TB. Water in the lungs was evaluated by measuring lung weight/TB ratio.

### Statistical analysis

Data from cardiac function and morphology *in vivo* and *in vitro*, basal isometric contraction, and western blot are reported as means±SD, and comparisons between groups were performed using the Student's *t*-test for independent samples. Inotropic maneuvers are reported as median, minimum, and maximum or as means±SD, using different methods of variance analysis for repeated measures. Muscle length was compared with RT and DT by the least squares method (39). The level of significance was 0.05. Normality test was performed.

## Results

*Post-mortem* (Table 1) and echocardiographic (Table 2) structural studies showed that the AoS group had a remodeled RV and AT. The LV had a concentric hypertrophy. Echocardiographic functional analysis (Table 3) showed an improvement in systolic function (EF, FS). Diastolic functional variables did not show any change compared to Sham. However, if we consider the fact that there was an increase in relative wall thickness and LA dimensions, it is possible to infer that LV had diastolic dysfunction.

**Table 1:** Post-mortem structural study of animals submitted to simulated surgery (Sham) and animals submitted to aortic stenosis surgery (AoS).

	Sham	AoS	P
BW (g)	470±36	479±73	0.74
TB (cm)	4.34±0.12	4.30±0.09	0.35
LV/TB	0.20±0.01	0.31±0.02	<0.001
RV/TB	0.055±0.004	0.061±0.008	0.04
AT/TB	0.023±0.002	0.04±0.01	<0.001
TH/TB	0.28±0.02	0.41±0.03	<0.001
LUNGS/TB	0.41±0.05	0.43±0.07	0.58

Data are reported as means ±SD (n=10/group). AoS *vs* Sham, Student's *t*-test. BW: body weight; TB: tibia length; LV: left ventricle; RV: right ventricle; AT: atria; TH: total heart.

**Table 2:** Structural study by echocardiogram.

	Sham	AoS	P
LVDD (mm)	8.64±0.46	7.76±0.29	<0.001
LVDD/TB (mm/cm)	1.99±0.10	1.81±0.06	<0.001
DPWT (mm)	1.50±0.11	1.90±0.22	<0.001
RWT	0.35±0.03	0.49±0.06	<0.001
LA (mm)	5.86±0.47	6.68±0.65	0.005
LA/TB (mm/cm)	1.35±0.10	1.56±0.16	0.003
LA/AO	1.49±0.17	1.68±0.21	0.04

Data are reported as means±SD (n=10/group). AoS *vs.* Sham, Student's *t*-test. Sham: control group submitted to surgery without clipping, AoS: aortic stenosis group submitted to surgery with clipping, n=10; LVDD: left ventricle diastolic diameter; TB: tibia length; DPWT: diastolic posterior wall thickness; RWT: relative wall thickness; LA: left atrium diameter; AO: aorta diameter.

**Table 3:** Functional study by echocardiogram of animals submitted to simulated surgery (Sham) and animals submitted to aortic stenosis surgery (AoS).

	Sham	AoS	P
HR (bpm)	243±12	273±25	0.003
EF (%)	0.86±0.02	0.93±0.04	<0.001
FS (%)	28.23±1.95	34.77±4.88	0.001
E (cm/s)	82.20±9.83	89.10±16.81	0.28
A (cm/s)	51.90±6.97	60.50±17.95	0.17
E/A	1.60±0.25	1.56±0.42	0.76
EDT (ms)	44.00±5.83	37.56±7.89	0.06
IVRT (ms)	26.60±2.99	23.50±4.55	0.09

Data are reported as means±SD (n=10/group). AoS *vs* Sham, Student’s *t*-test. HR: heart rate; EF: ejection fraction; FS: midwall fraction shortening; E/A: ratio between filling flow peak (E wave) and atrial contraction flow peak (A wave); EDT: E wave deceleration time; IVRT: left ventricular isovolumetric relaxation time.

**Figure 1:**
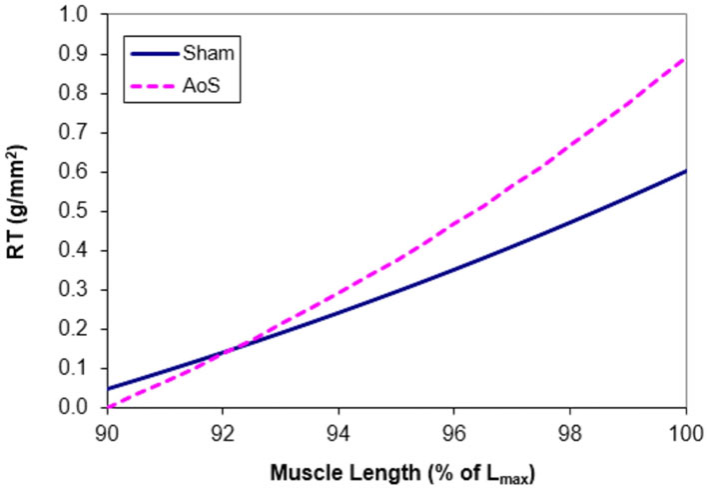
Relationship between muscle length variation, in % of maximum length (L_max_), and resting tension (RT). P<0.01, Sham *vs* aortic stenosis (AoS) (non-linear regression model adjustment using the least squares method).

**Figure 2:**
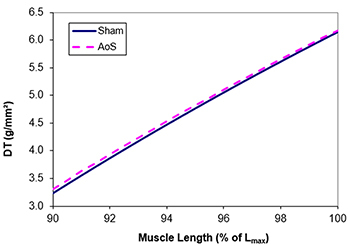
Relationship between muscle length variation, in % of maximum length (L_max_), and developed tension (DT). AoS: aortic stenosis.

*In vitro* papillary muscle functional study at basal condition (Table 4) showed increased RT in AoS muscle compared to the Sham group. This behavior was also seen in the relationship between RT and muscle length variation (Figure 1). These data showed that hypertrophied muscle is more rigid than the control, suggesting diastolic dysfunction, which corroborates the assumption from the echocardiographic study. Systolic function remained the same in both groups (Table 4 and Figure 2), which differs from the *in vivo* study, where it showed an improvement in the hypertrophied heart. TPT was increased in AoS animals.

**Table 4:** Basal isometric contraction of animals submitted to simulated surgery (Sham) and animals submitted to aortic stenosis surgery (AoS).

	Sham	AoS	P
DT (g/mm^2^)	5.91±0.98	6.20±1.66	0.65
RT (g/mm^2^)	0.64±0.18	0.86±0.21	0.02
+dT/dt (g/mm^2^ per s)	68.70±12.64	62.37±14.25	0.31
-dT/dt (g/mm^2^ per s)	26.35±4.26	27.49±8.13	0.70
TPT (ms)	166±21	192±14	0.005
CSA (mm^2^)	1.06±0.21	1.20±0.22	0.18

Data are reported as means±SD (n=10/group). AoS *vs* Sham; Student's *t*-test. Extracellular calcium concentration: 2.5 mM. DT: maximum developed tension; RT: resting tension; +dT/dt: maximum rate of tension development; -dT/dt: maximum rate of tension decline; TPT: time-to-peak tension; CSA: cross-sectional area.

**Figure 3:**
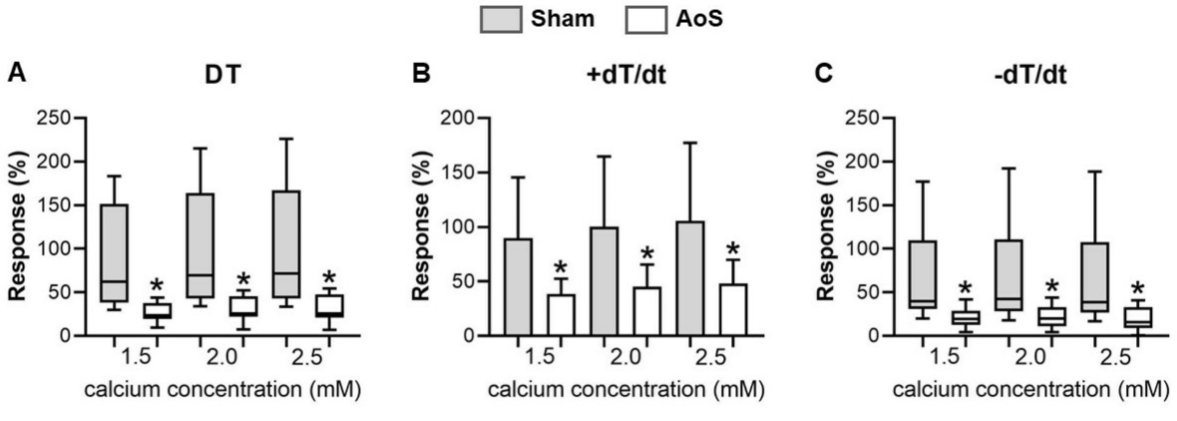
Percent calcium elevation from baseline (Ca^2+^ concentration: 0.5 mM). DT: maximum developed tension; +dT/dt: maximum rate of tension development; -dT/dt: maximum rate of tension decline. Sham: animals submitted to simulated surgery (n=10); AoS: animals submitted to aortic stenosis surgery (n=10). Data are reported as median, maximum, and minimum (**A** and **C**), or means±SD (**B**). *P<0.05, Sham *vs* AoS [analysis of variance for repeated measures and Bonferroni *post hoc* test (bar chart) or Kruskall Wallis and Wilcoxon tests complemented by Dunn *post hoc* test (box plot)].

Calcium elevation effects are shown in Figure 3. This maneuver revealed a significant difference between the two groups, although there is no distinction between moments in each group. AoS had significantly less response to increased extracellular Ca^2+^ concentration compared to controls.

**Figure 4:**
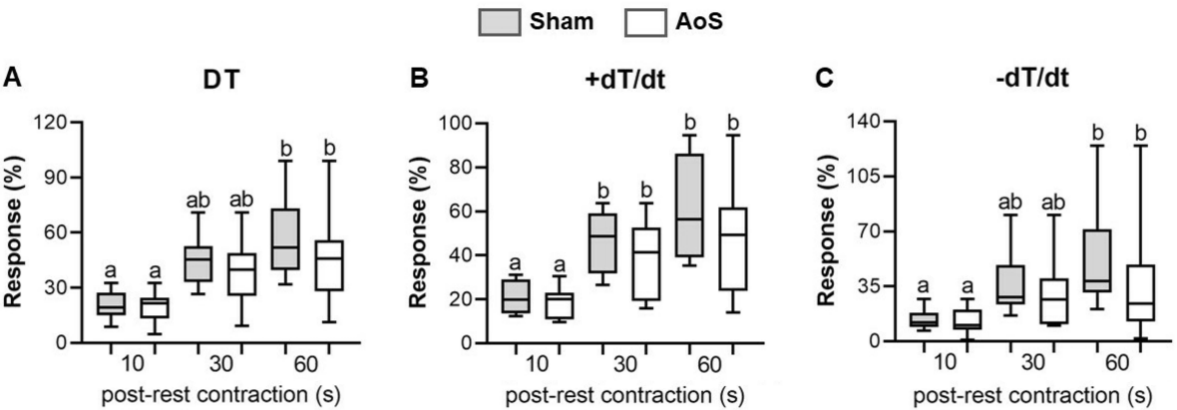
Percent of post-rest contraction from baseline (Ca^2+^ concentration: 0.5 mM). DT: maximum developed tension; +dT/dt: maximum rate of tension development; -dT/dt: maximum rate of tension decline. Sham: animals submitted to simulated surgery (n=10); AoS: animals submitted to aortic stenosis surgery (n=10). Data are reported as median, maximum, and minimum. P<0.05, Sham *vs* AoS (Kruskall Wallis and Wilcoxon tests complemented by Dunn *post hoc* test). Different letters indicate statistical difference among the moments within each group.

Differently from the Ca^2+^ elevation maneuver, no divergence in post-rest contraction was seen between groups (Figure 4), only between different moments. Although DT and -dT/dt increased from moment 10" to 60", +dT/dt was statistically divergent after 30" in both groups.

**Figure 5:**
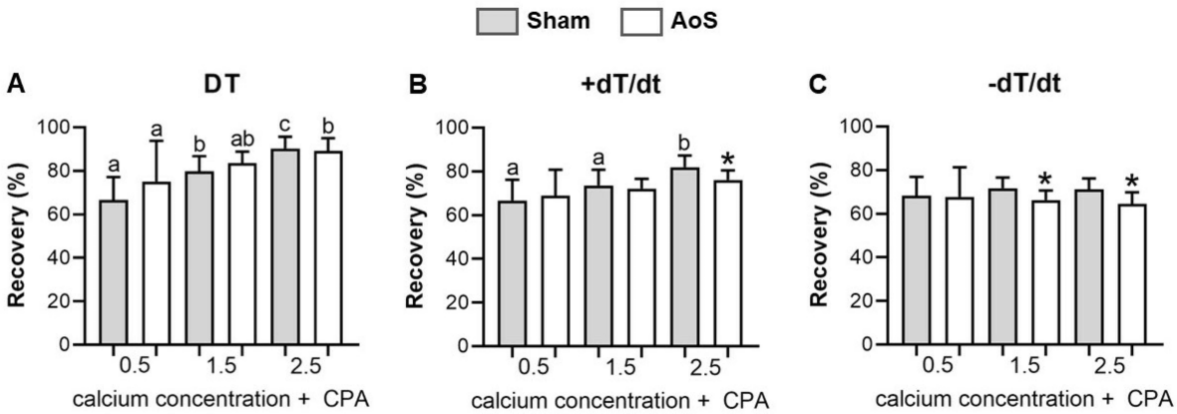
Function recovery in percentage post cyclopiazonic acid (CPA). DT: maximum developed tension; +dT/dt: maximum rate of tension development; -dT/dt: maximum rate of tension decline. Sham: animals submitted to simulated surgery (n=10); AoS: animals submitted to aortic stenosis surgery (n=10). Data are reported as means±SD. *P<0.05, AoS *vs* Sham (analysis of variance for repeated measures and Bonferroni *post hoc* test). Different letters indicate statistical difference among the moments within each group.

CPA blockage (Figure 5), which is also used to analyze SERCA2a behavior, showed a difference between AoS and Sham groups for +dT/dt (2.5 mM) and -dT/dt (1.5 and 2.5 mM), which differed from the post-rest contraction study. Hypertrophied muscle presented lower contraction and relaxation speeds than control rats at these same moments in SERCA2a blockage maneuver.

**Figure 6:**
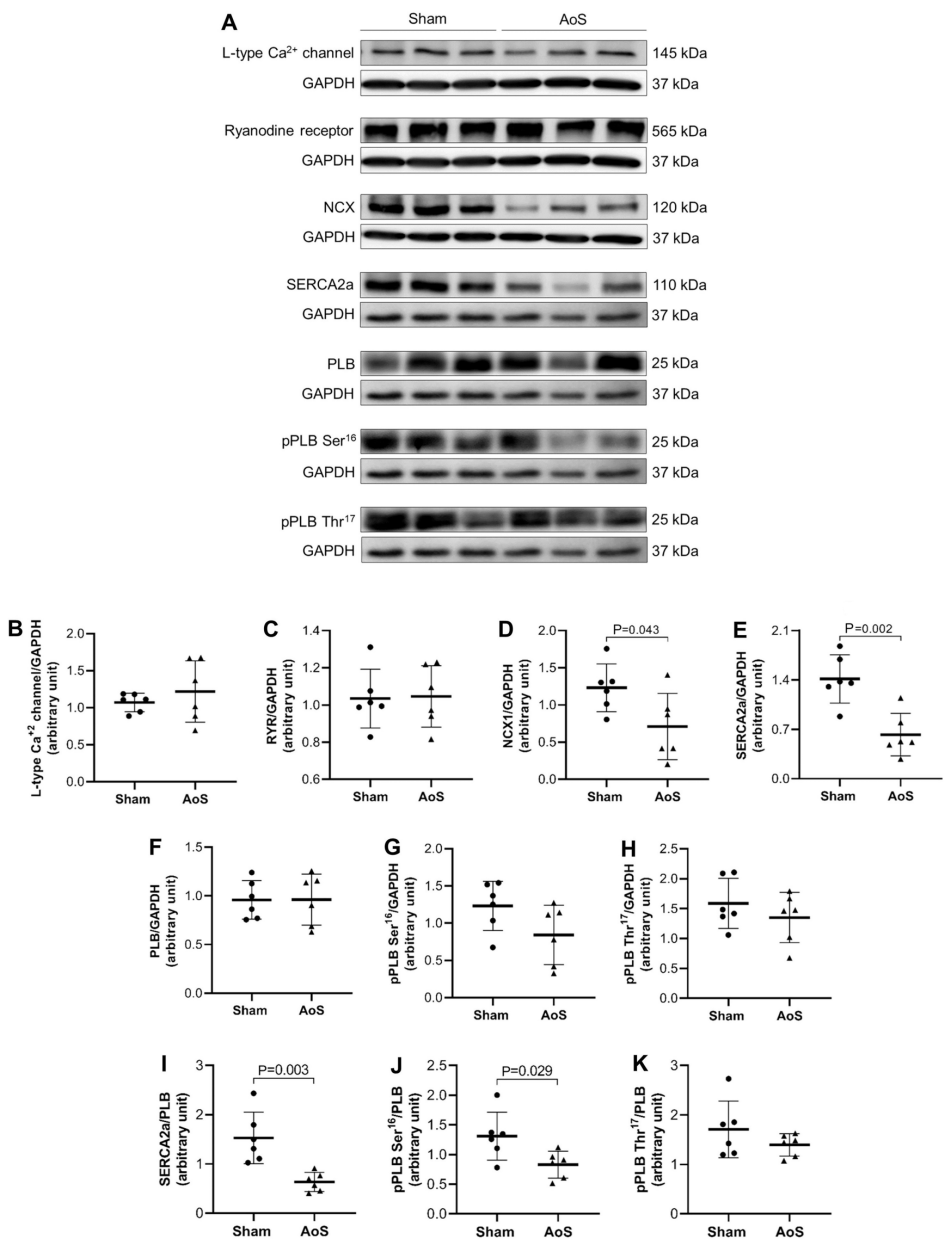
Western blot analysis of intracellular Ca^2+^-cycling proteins in myocardium from Sham and aortic stenosis (AoS) rats (n=6 in each group). **A,** Representative bands of the proteins. Quantification of myocardial (**B**) L-Type Ca^2+^ channel, (**C**) ryanodine receptor (RyR), (**D**) Na^+^/Ca^2+^ exchanger (NCX1), (**E**) sarcoplasmic reticulum calcium-ATPase (SERCA2a), (**F**) total phospholamban (PLB), (**G**) phosphorylated PLB on serine-16 (pPLB Ser16), and (**H**) phosphorylated PLB on threonine-17 (pPLB Thr17) normalized to glyceraldehyde 3-phosphate dehydrogenase (GAPDH). Quantification of (**I**) SERCA2a, (**J**) pPLB Ser16, and (**K**) pPLB Thr17 normalized to total PLB. Data are reported as means±SD. P<0.05, Student's *t*-test.

To complement Ca^2+^ intracellular transient analysis, we investigated the expression of proteins involved in this phenomenon. Of the studied parameters (Figure 6), only NCX1 and SERCA2a showed decreases in hypertrophied heart compared to controls. SERCA2a/PLB and phosphorylated PLB serine 16/PLB ratios were also reduced. These data supported the hypothesis that the proteins related to intracellular Ca^2+^ homeostasis were affected in hearts under pressure overload.

## Discussion

It is known that when the heart is submitted to a pressure overload, such as aortic banding, its response is enhanced contractile strength, generating a thicker ventricle wall and smaller chamber diameter. In its early stages, this process helps to maintain the correct operation of the cardiovascular system, however, over time it will damage the heart and its function will fail (11,12,25).

Our echocardiography showed that after 18 weeks of aortic stenosis animals presented concentric hypertrophy, diastolic dysfunction, and increased systolic function. This result was in agreement with literature that shows enhanced systolic function after 18 weeks (12). On the other hand, Mendes et al., using the same methodology and observation time, found an initial decrease after 18 weeks (1) in AoS. Although our study found no statistical difference between the variables that analyze diastolic function in echocardiogram, the increase in relative wall thickness implies in hypertrophy, which impairs muscle energy balance, jeopardizing diastole, and increasing end diastolic pressure, which ends up leading to left atrium enlargement, also found in our study. Literature shows that diastolic dysfunction is usually found in this experimental cardiopathy model after 6 weeks of aortic stenosis (11,12,25).

We studied isolated papillary muscle to verify whether the alterations in hypertrophied muscle seen *in vivo* were still present even under controlled conditions such as *in vitro* preload, afterload, and heart rate. We also analyzed whether the calcium transient misbalance could be one of the mechanisms involved in this experimental heart failure model.

The data were partially compatible with echocardiogram. RT was elevated, both at basal condition and during muscle length variation, proving that AoS animals had lower heart muscle complacency, which indicates diastolic dysfunction. According to Silveira et al. (11), one factor that could explain this finding is an increase in cytosolic Ca^2+^ at the end of the diastole, leaving ions bound to C troponin.

However, although the image exam showed enhanced systolic function, DT in this study was similar in both groups. Higher TPT in AoS could indicate the beginning of mechanical systolic dysfunction, which was not detected in the echocardiogram. This behavioral distinction is probably due to the fact that papillary muscle is analyzed in situations with controlled load and heart rate and is free from neurohumoral variations, present in *in vivo* heart.

The inotropic maneuvers used in our papillary muscle study showed that dysfunctional hypertrophied myocardium may be related to calcium-handling alterations. Thus, elevated extracellular calcium concentrations displayed a robust difference in behavior between groups, with aortic stenosis animals presenting less response to an increase in this ion. This pronounced change did not appear in post-rest contraction maneuver but was present at a lower level in SERCA2a blockage with cyclopiazonic acid. These results suggested a dysfunction in Ca^2+^ entry through L-type Ca^2+^ channels, although expression was normal in the molecular study. There was also less damage of the intracellular Ca^2+^ cycle in the myocardium, which could be compatible with decreased SERCA2a and SERCA2a/PLB expression.

The decrease in NCX1 expression, the most important mechanism involved in calcium efflux across the plasma membrane, would allude to enhanced intracellular Ca^2+^ in hypertrophied muscle, which corroborates the idea that remaining ions cause the diastolic dysfunction shown in IPM through an elevation in resting tension.

An interesting review looked at Ca^2+^-handling proteins and their relationship with hypertrophied muscle (40). In several studies, Locatelli et al. (40) found that NCX1 is usually enhanced in heart failure and hypertrophy and that SERCA expression is reduced or regulator levels are increased. This review only corroborates all the divergences between studies and between data within studies that are cited in the present article. Perhaps variables such as similarity between animals, experiment conditions, and evaluator performances have a huge influence on results; that is why scientific research needs to continue in its quest for an answer.

In conclusion, data in our study showed that 18 weeks after AoS surgery rats presented diastolic dysfunction, which could be related to changes in calcium-homeostasis behavior.

## References

[1] Mendes OC, Campos DHS, Damatto RL, Sugizaki MM, Padovani CR, Okoshi K (2010). Cardiac remodeling: serial analysis and indexes for early detection of ventricular dysfunction [in Portuguese]. Arq Bras Cardiol.

[2] Cicogna AC, Okoshi MP, Okoshi K (2000). História natural da remodelação miocárdica: da agressão aos sintomas [in Portuguese]. Rev Soc Cardiol do Estado de São Paulo.

[3] Boluyt MO, Robinson KG, Meredith AL, Sem S, Lakatta EG, Crow MT (2005). Heart failure after long-term supravalvar aortic constriction in rats. Am J Hypertens.

[4] Okoshi MP, Matsubara LS, Franco M, Cicogna AC, Matsubara BB (1997). Myocyte necrosis is the basis for fibrosis in renovascular hypertensive rats. Braz J Med Biol Res.

[5] Mestrinel MA (2003). Avaliação morfológica, bioquímica e funcional do remodelamento cardíaco desencadeado por sobrecarga pressórica em ratos com e sem insuficiência cardíaca congestiva [Thesis; in Portuguese]. Botucatu: Faculdade Medicina, Universidade Estadual Paulista.

[6] Ribeiro HB, Okoshi K, Cicogna AC, Bregagnollo EA, Rodrigues MAM, Padovani CR (2003). Estudo evolutivo da morfologia e função cardíaca em ratos submetidos a estenose aórtica supravalvar [in Portuguese]. Arq Bras Cardiol.

[7] Matsui H, MacLennan DH, Alpert NR, Periasamy M (1995). Sarcoplasmic reticulum gene expression in pressure overload-induced cardiac hypertrophy in rabbit. Am J Physiol.

[8] Rockman HA, Ono S, Ross RS, Jones LR, Karimi M, Bhargava V (1994). Molecular and physiological alterations in murine ventricular dysfunction. Proc Natl Acad Sci USA.

[9] Tsutsui H, Ishibashi Y, Imanaka-Yoshida K, Yamamoto S, Yoshida T, Sugimachi M (1997). Alterations in sarcoplasmic reticulum calcium-storing proteins in pressure-overload cardiac hypertrophy. Am J Physiol.

[10] De la Bastie D, Levitsky D, Rappaport L, Mercadier JJ, Marotte F, Wisnewsky C (1990). Function of the sarcoplasmic reticulum and expression of its Ca2 (+)-ATPase gene in pressure overload-induced cardiac hypertrophy in the rat. Circ Res.

[11] Silveira CFSMP, Campos DHS, Freire PP, Deus AF, Okoshi K, Padovani CR (2017). Importance of SERCA2a on early isolated diastolic dysfunction induced by supravalvular aortic stenosis in rats. Braz J Med Biol Res.

[12] De Tomasi LC, Campos DHS, Sant'Ana PG, Okoshi K, Padovani CR, Murata GM (2018). Pathological hypertrophy and cardiac dysfunction are linked to aberrante endogenous unsaturated fatty acid metabolism. PLoS One.

[13] Hiranandani N, Raman S, Kalyanasundaram A, Periasamy M, Janssen PML (2007). Frequency-dependent contractile strength in mice over- and underexpressing the sarco (endo)plasmic reticulum calcium-ATPase. Am J Physiol Regul Integr Comp Physiol.

[B14] Periasamy M, Huke S (2001). SERCA pump level is a critical determinant of Ca (2+)homeostasis and cardiac contractility. J Mol Cell Cardiol.

[15] Arai M, Alpert NR, MacLennan DH, Barton P, Periasamy M (1993). Alterations in sarcoplasmic reticulum gene expression in human heart failure. A possible mechanism for alterations in systolic and diastolic properties of the failing myocardium. Circ Res.

[16] Freire PP, Alves CAB, Deus AF, Leopoldo APL, Leopoldo AS, Silva DCT (2014). Obesidade não Acarreta Desequilíbrio entre Fosforilação e Desfosforilação da Fosfolambam Miocárdica. Arq Bras Cardiol.

[17] Tanaka N, Dalton N, Mao L, Rockman HÁ, Peterson KL, Gottshall KR (1996). Transthoracic echocardiography in models of cardiac disease in the mouse. Circulation.

[B18] Feldman AM, Weinberg EO, Ray PE, Lorell BH (1993). Selective changes in cardiac gene expression during compensated hypertrophy and the transition to cardiac decompensation in rats with chronic aortic banding. Circ Res.

[19] Miyamoto MI, del Monte F, Schmidt U, DiSalvo TS, Kang ZB, Matsui T (2000). Adenoviral gene transfer of SERCA2a improves left-ventricular function in aortic-banded rats in transition to heart failure. Proc Natl Acad SCi USA.

[20] Holtz J (1993). Myocardial hypertrophy after myocardial infarct: what is the significance of phenotype changes in cardiocytes? [in German]. Herz.

[21] Carnicelli V, Frascarelli S, Ghelardoni S, Ronca-Testoni S, Zucchi R (2008). Short-term effects of pressure overload on the expression of genes involved in calcium homeostasis. Mol Cell Biochem.

[22] Røe AT, Aronsen JM, Skardal K, Hamdani N, Linke WA, Danielsen HE (2017). Increased passive stiffness promotes diastolic dysfunction despite improved Ca21 handling during left ventricular concentric hypertrophy. Cardiovasc Res.

[23] Rouhana S, Farah C, Roy J, Finan A, Araujo GR, Bideaux P (2019). Early calcium handling imbalance in pressure overload-induced heart failure with nearly normal left ventricular ejection fraction. Biochim Biophys Acta Mol Basis Dis.

[24] Hadipour-Lakmehsari S, Driouchi A, Lee SH, Kuzmanov U, Callaghan NI, Heximer SP (2019). Nanoscale reorganization of sarcoplasmic reticulum in pressureoverload cardiac hypertrophy visualized by dSTO RM. Sci Rep.

[25] Mendes OC, Sugizaki MM, Campos DS, Damatto RL, Leopoldo AS, Lima-Leopoldo AP (2013). Exercise tolerance in rats with aortic stenosis and ventricular diastolic and/or systolic dysfunction. Arq Bras Cardiol.

[B26] Gonçalves G, Zornoff LAM, Ribeiro HB, Okoshi MP, Cordaro FRS, Okoshi K (2005). Blockade of renin-angiotensin system attenuates cardiac remodeling in rats undergoing aortic stenosis [in Portuguese]. Arq Bras Cardiol.

[B27] Okoshi K, Ribeiro HB, Okoshi MP, Matsubara BB, Gonçalves G, Barros R (2004). Improved systolic ventricular function with normal myocardial mechanics in compensated cardiac hypertrophy. Jpn Heart J.

[28] Carvalho RF, Cicogna AC, Campos Ge, De Assis JM, Padovani CR, Okoshi MP (2003). Myosin heavy chain expression and atrophy in rat skeletal muscle during transition from cardiac hypertrophy to heart failure. Int J Exp Pathol.

[29] Cicogna AC, Padovani CR, Georgette JC, Aragon FF, Okoshi MP (1999). Efeito da restrição protéico-calórica sobre a função mecânica dos músculos cardíacos hipertrofiados. Arq Bras Cardiol.

[B30] Guazzi M, Brenner DA, Apstein CS, Saupe KW (2001). Exercise intolerance in rats with hypertensive heart disease is associated with impaired diastolic relaxation. Hypertension.

[B31] de Paiva SA, Zornoff LA, Okoshi PM, Okoshi K, Matsubara LS, Matsubara BB (2003). Ventricular remodeling induced by retinoic acid supplementation in adult rats. Am J Physiol Heart Circ Physiol.

[B32] Bayat H, Swaney JS, Ander AN, Dalton N, Kennedy BP, Hammond HK (2002). Progressive heart failure after myocardial infarction in mice. Basic Res Cardiol.

[B33] Sjaastad I, Sejersted OM, Ilebekk A, Bjornerheim R (2000). Echocardiographic criteria for detection of postinfarction congestive heart failure in rats. J Appl Physiol.

[B34] Ono K, Masuyama T, Yamamoto K, Doi R, Sakata Y, Nishikawa N (2002). Echo Doppler assessment of left ventricular function in rats with hypertensive hypertrophy. J Am Soc Echocardiogr.

[35] Cantor EJF, Babick AP, Vasanji Z, Dhalla NS, Netticadan T (2005). A comparative serial Echocardiographic analysis of cardiac structure and function in rats subjected to pressure or volume overload. J Mol Cell Cardiol.

[36] Cicogna AC, Robinson KG, Conrad CH, Squire R, Okoshi MP, Bing OH (1997). Role of myocardial contractile status and relaxation in ventricular dysfunction during the transition of heart hypertrophy to failure [in Portuguese]. Arq Bras Cardiol.

[B37] Vileigas DF, Deus AF, Silva DCT, Tomasi LC, Campo DHS, Adorni CS (2016). Saturated high-fat diet-induced obesity increases adenylate cyclase of myocardial b-adrenergic system and does not compromise cardiac function. Physiol Rep.

[38] Leopoldo AS, Lima-Leopoldo AP, Sugizaki MM, do Nascimento AF, de Campos DH, Luvizotto RAM (2011). Involvement of L-type calcium channel and SERCA2a in myocardial dysfunction induced by obesity. J Cell Physiol.

[39] Draper NR, Smith H (1998). Applied regression analysis. 3rd ed.

[40] Locatelli J, Assis LVM, Isoldi MC (2014). Calcium handling proteins: structure, function, and modulation by exercise. Heart Fail Rev.

